# Assessment of neutrophil/lymphocyte ratio and mean platelet volume values in patients with diabetes mellitus diagnosis: A case–control study

**DOI:** 10.1097/MD.0000000000039661

**Published:** 2024-09-13

**Authors:** Kemal Aygün, Ayça Asma Sakalli, Halime Seda Küçükerdem, Olgu Aygün, Özden Gökdemir

**Affiliations:** a Department of Hematology, Izmir Katip Celebi University, Atatürk Training and Research Hospital, Izmir, Turkey; b Department of Family Medicine, Balikesir Atatürk City Hospital, Balikesir, Turkey; c Department of Family Medicine, Izmir City Hospital, Izmir, Turkey; d Department of Family Medicine, Izmir University of Economics, Izmir, Turkey.

**Keywords:** erythrocyte indices, glycated hemoglobin, glycemic control, mean platelet volume, neutrophils

## Abstract

Diabetes mellitus, fundamentally characterized by hyperglycemia, leads to significant metabolic disturbances. Type 2 diabetes mellitus is a chronic, inflammatory, preventable metabolic disease that is a significant health issue globally. The neutrophil-to-lymphocyte ratio (NLR) is an essential marker of systemic inflammation. We aimed to reveal the relationship between long-term glucose control and NLR, mean platelet volume (MPV), and red blood cell width in patients with type 2 diabetes. This was a retrospective case–control study. A total of 3532 applications in 2 years time were identified. Age, gender, medical history, white blood cell (WBC), hemoglobin, mean corpuscular volume (MCV), MPV, red blood cell width, NLR, hematocrit, platelet, C-reactive protein, Haemoglobin A1C data of the patients were analyzed. 1790 patients were included. A significant positive correlation was found between HbA1c and age, white blood cell, hematocrit, MCV, red blood cell width, NLR, and CRP. A statistically significant negative correlation was found between HbA1c and MCV. The results showed statistically significant differences between NLR, MPV, WBC, MCV, age, and HbA1c levels. Increased HbA1c levels are usually associated with an increase in these parameters. This is important for determining the risk of complications and protecting target organs in diabetic patients. A significant decrease in MCV levels was found as HbA1c levels increased. This suggests that evaluating red blood cells in routine controls of diabetic patients may indicate glycemic control. These findings may be valuable in early diagnosis of complications.

## 1. Introduction

Diabetes mellitus, fundamentally characterized by hyperglycemia, is a condition leading to significant metabolic disturbances. Type 2 diabetes mellitus (T2DM) is a chronic, inflammatory, preventable metabolic disease that is a significant health issue globally. Although it can effectively reduce complications when detected early, patients are often known to have been living with the disease for some time when diagnosed. According to the International Diabetes Federation, the number of people affected by diabetes mellitus was surprisingly estimated to be 537 million in 2021, with the potential to increase by 19.7% by 2030. As a result, it is expected that more than 10% of the world’s population will be affected by diabetes mellitus in the coming years.^[1,2]^

T2DM, also known as non-insulin-dependent or adult-onset diabetes, is a form that constitutes the majority of diabetic patients. This type is typically characterized by insulin resistance and relative insulin deficiency. It arises from a combination of genetic factors, aging, obesity, overnutrition, lack of exercise, and various environmental factors such as stress. T2DM is a disease that develops as a result of the convergence of multiple genetic and environmental factors. Although its exact etiology may not be fully understood, it is generally based on multiple causes. Autoimmune destruction usually does not occur in β-cells in developing this type of diabetes.^[1–3]^

T2DM is considered a serious disease and can lead to macrovascular complications, such as cerebrovascular disease, coronary artery disease, and peripheral artery disease, as well as microvascular complications, including nephropathy, retinopathy, and neuropathy.^[4]^ In T2DM, hyperglycemia and insulin resistance are associated with stimulating pro-inflammatory processes. Proinflammatory responses induced by various immune cells lead to low-grade inflammation. Adiposity and obesity are often contributing factors to the risk of T2DM, and inflammatory responses are associated with the complications and progression of T2DM.^[5]^

The neutrophil-to-lymphocyte ratio (NLR) is an essential marker of systemic inflammation. Furthermore, NLR is an indicative marker in various malignancies and acute coronary syndrome. NLR indicates systemic inflammation in chronic kidney disease and diabetic nephropathy. It is easily measured and relatively cost-effective. Furthermore, NLR may be increased in patients with T2DM, which may indicate the inflammatory burden of T2DM. This study aimed to reveal the relationship between NLR and glucose control in patients with T2DM.We aimed to reveal the relationship between long-term glucose control (HbA1C) and NLR, mean platelet volume (MPV), red blood cell width (RDW) in patients with type 2 DM.^[3,5–7]^

## 2. Materials and methods

### 2.1. Study population and data collection

This study was structured as a retrospective case–control study. It followed the principles of the Declaration of Helsinki, and approval was obtained from the ethics committee of Izmir Bozyaka Training and Research Hospital.

Records of patients who presented to the family medicine district outpatient clinic of a training and research hospital between 2021 and 2023 for various reasons were retrospectively evaluated. About 3532 patients were identified.

### 2.2. Laboratory

Age, gender, medical history, white blood cell (WBC), hemoglobin (Hgb), mean corpuscular volume (MCV), MPV, red blood cell width (RDW), NLR, Haematocrit (HCT), platelet (PLT), C-reactive protein (CRP), Haemoglobin A1C (HbA1c) data of the patients were analyzed. Haemoglobin A1C (HbA1c) was used to monitor blood glucose control. Patients were divided into diabetic case group (HbA1c > 6.5%), prediabetic case group (HbA1c: 5.7–6.5%), and nondiabetic control group (HbA1c < 6.5%).^[8]^

### 2.3. Inclusion and exclusion criteria

About 133 patients under 18 years of age, 3 pregnant women, 70 patients without hemogram data, 91 patients without CRP data, and 187 patients without HbA1c data were excluded from the study. Total 249 patients with a previous diagnosis of diabetes made by a physician or those taking insulin or oral hypoglycemic agents, uncontrolled or secondary hypertension, hematological disease (such as essential thrombocytosis and beta thalassemia) cirrhosis, heart failure, kidney failure, using steroids, or with a history of malignancy, acute infections such as acute upper respiratory infections, leukopenia (WBC < 4 × 10^9^/L) or leukocytosis (WBC > 11 10^9^/L), anemia (females with Hgb < 12 g/dL, males with Hgb < 13.5 g/dL), polystemia (females with Hgb > 16 g/dL, males with Hgb > 16.5 g/dL), abnormal C-reactive protein levels (Normal level of CRP in our lab is 0–5 mg/L) were excluded from the study.^[9–11]^

**Figure 1.**
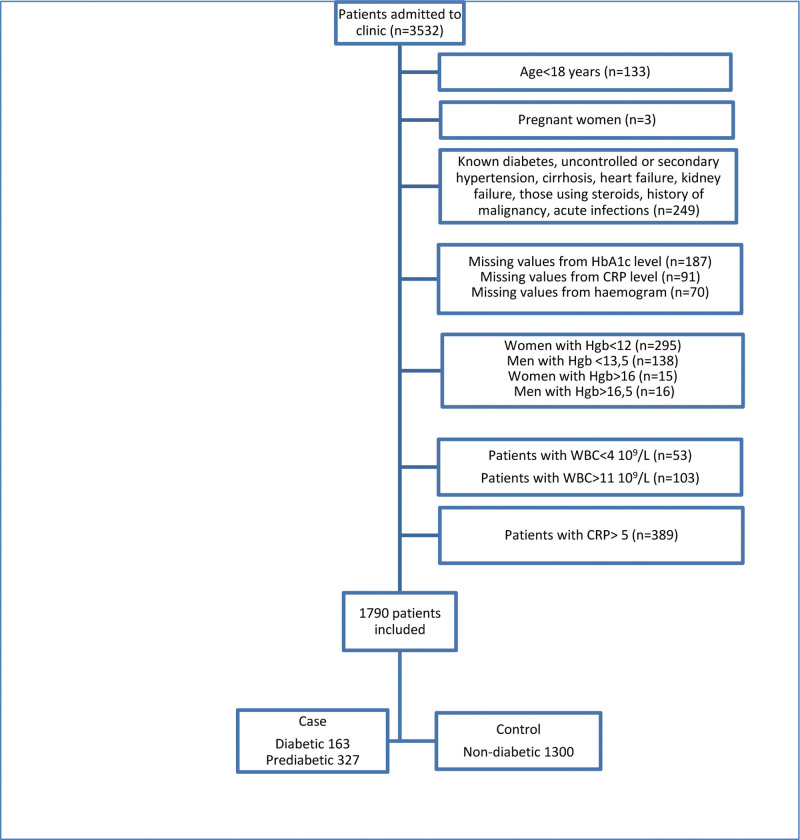
Material-method diagram.

A total of 1790 patients were included in the study (Fig. 1).

### 2.4. Statistical method

In the statistical analysis of the data, the SPSS 25.0 package program was used. Categorical measurements were summarized as numbers and percentages, while continuous measurements were summarized as mean and standard deviation (median and minimum–maximum where necessary). For comparing categorical variables, the Chi-square test or Fisher test statistics were used. For comparing continuous measurements among groups, distributions were checked, and depending on the number of variables, 1-way analysis of variance (ANOVA) or Student *t* test were used for parameters showing parametric distribution, while the Kruskal–Wallis test or Mann–Whitney U test were used for parameters not showing parametric distribution.

The correlation between variables was determined using Spearman correlation coefficient. The evaluation of the correlation coefficient is as follows: if *r* ≥ 0.91, there is a high correlation between variables; if 0.90 ≤ *r* ≥ 0.71, the correlation between variables is good; if 0.70 ≤ *r* ≥ 0.51, the correlation between variables is moderate; if 0.50 ≤ *r* ≥ 0.31, the correlation between variables is low; if *r* ≤ 0.3, there is no correlation between variables.

In the study, for determining the cutoff value of HbA1c in patients with and without DM, sensitivity and specificity values were calculated, and the area under the ROC curve was evaluated using ROC Analysis. A significance level of 0.05 was considered for all tests.

## 3. Results

28.3% of the participants were male and 71.7% were female. The mean age was 57.26 years. The mean WBC is 7.32 10^9^/L. The mean red blood cell (RBC) is 4.8 10 × 6/uL. The mean Hgb is 14.01 g/dL. HCT average is 42.5. The platelet count (PLT) average is 274.11 10^9^/L. The MCV average is 88.75 fL. MPV average is 10.68 fL. RDW-CV average is % 13.68. NLR mean is 1.76. According to HbA1c groups, the findings of a comparison of nondiabetic, prediabetic, and diabetic groups with other parameters are shown in Table 1.

**Table 1:** Comparison of quantitative data according to HbA1c groups.

	Nondiabetic: HbA1c < 6% (n = 1300)	Prediabetic: HbA1c = 6 to 6.4% (n = 327)	Diabetic: HbA1c ≥ 6.5% (n = 163)	Total (n = 1790)	Test statistic	*P*
Age (yr)	50 (18–88)a	63 (21–92)b	66 (23–88)b	55 (18–92)	248,392	<.001*
WBC (10^9^/L)	6.65 (4.02–11)a	7.22 (4.01–10.78)b	7.43 (4.1–10.82)b	6.82 (4.01–11)	53,981	<.001*
Hgb (g/dL)	13.8 (12–16.5)	13.8 (11.9–16.4)	14 (12–16.4)	13.9 (11.9–16.5)	2041	.361*
HCT	41.8 (34–51.9)	42 (34.7–50.5)	42.4 (35.5–49.9)	41.9 (34–51.9)	2347	.309*
PLT (10^9^/L)	266 (114–513)	273 (113–507)	258 (154–443)	266 (113–513)	1936	.380*
MCV (fL)	88.9 (11.4–104.6)	88.8 (64.2–99.5)	88.1 (10.9–99.3)	88.9 (10.9–104.6)	5296	.071*
MPV (fL)	10.6 (8.1–14)	10.7 (8.1–13.7)	10.7 (8–14.1)	10.6 (8–14.1)	1792	.408*
RDW-CV (%)	13 (11.1–45.4)a	13.4 (11.7–32.5)b	13.4 (11.4–45.2)b	13.2 (11.1–45.4)	73,963	<.001*
NLR	1.53 (0.46–9.47)	1.48 (0.61–4.25)	1.64 (0.46–5.3)	1.52 (0.46–9.47)	5813	.055*
CRP (mg/dL)	1.3 (0–4.99)a	1.74 (0.17–4.9)b	1.8 (0.2–4.8)b	1.42 (0–4.99)	41,282	<.001*
HbA1c (%)	5.5 (4–5.9)a	6.1 (6–6.4)b	6.9 (6.5–18)c	5.7 (4–18)	1097,937	<.001*

* Kruskal–Wallis *H* test.

a^,b,^c There is no difference between groups with the same letter.

The relationship between age, WBC, HCT, MCV, MPV, RDW-CV, NEU/LENF, CRP, and HbA1c was statistically significant for Spearman correlation value (Table 2).

**Table 2:** The correlation between HbA1c and variables.

Variables	HbA1c	
	*r*	*P*
Age	0.486*	**<.001**
WBC	0.198*	**<.001**
HGB	0.003*	.917
HCT	0.064*	**.007**
PLT	0.041*	.087
MCV	−0.067*	**.005**
MPV	0.06*	**.011**
RDW-CV	0.254*	**<.001**
NEU/LENF	0.18*	<**.001**
CRP	0.208*	**<.001**

Values with a significance level of *P* < .050 are shown in bold.

* Spearman.

## 4. Discussion

Today, almost half of people with diabetes mellitus are aged 65 and over. Age-related decline in the proliferation and replication potential of beta cells has been suggested as a reason for the increased prevalence of T2DM with increasing age.^[12]^ In our study, the relationship between HbA1c and age was found to be statistically significant with Spearman correlation value. Dubowitz et al found that “Aging is associated with increasing HbA1c levels.”^[13]^ Similarly, studies are showing that HbA1c levels are positively correlated with age.^[14,15]^ Establishing age-related reference values for HbA1c and applying them in daily practice could improve patient care and diabetes diagnosis.^[16]^

One of the mechanisms involved in the pathogenesis of diabetic vascular complications is the secretion of inflammatory cytokines and transcription factors by WBCs activated by advanced glycation end products, oxidative stress, angiotensin II, and cytokines.^[17]^ A study with Mendelian randomization showed that WBC count may have a positive causal association with T2DM risk.^[18]^ On the other hand, another Mendelian study showed that WBC was not associated with HbA1c.^[19]^ The present study observed a statistically significant positive, very weak correlation between HbA1c and WBC. As HbA1c levels increase, there may be an increase in WBC levels. In recent research, similar results have been reported.^[20–22]^ The WBC count, which has lower applicability and cost than other inflammatory markers, may guide clinical practice.

Our study found a significant positive correlation between HbA1c and MPV. It has been reported that platelet function and morphology are altered, platelet volume is larger, and platelet activity is increased due to hyperglycemia in diabetic patients.^[23]^ Platelet activation may contribute to the development of atherothrombosis and acute major arterial events. Studies in the literature show a positive correlation between HbA1c and MPV.^[24–26]^

Many previous studies have reported a high correlation between HbA1c and CRP levels.^[27]^ Our study found a significant positive correlation between HbA1c and CRP. Tong et al found that elevated CRP was associated with future diabetes development in a Norwegian adult population sample.^[28]^ According to Cheng et al, low CRP levels are associated with a high rate of regression from prediabetes to normoglycemia and a reduced likelihood of progression to diabetes. To prevent the patient from complications and to protect the target organs that high glucose and inflammation destroy, these parameters could enlighten the follow-up procedures.^[29,30]^

Studies show a significant correlation between HbA1c and MCV.^[31,32]^ This study found a statistically significant negative correlation between HbA1c and MCV. In addition, a significant difference was found in MCV values according to controlled and uncontrolled diabetes groups. However, there is also the view that poor glycemic control may increase MCV by causing membrane proteins to be glycosylated, displacing sodium and chloride ions near the cells.^[33]^

In the present study, a significant relationship has been identified between HbA1c levels and the NLR. This finding is similar to previous studies.^[34,35]^ Palella et al revealed that NLR was higher in patients with higher HbA1c levels and that NLR is a helpful prognostic marker that can predict vascular complications in T2DM.^[36]^ It is interesting that Singh et al declared that the observed elevation in NLR was consistent with higher HbA1c levels and corresponded to an intensified index of stroke severity.^[37]^ There is also a view that NLR may be related to transient glycemic indices rather than long-term variations in glycemic metabolism, such as HbA1c, as it reflects an acute response.^[38]^ In the present study, no relationship was found between NLR in the controlled diabetes group and the uncontrolled diabetes group. However,a meta-analysis by Adane et al showed that high NLR was significantly associated with poor glycemic control in T2DM patients (OR = 1.50, 95% CI: 1.30–1.93).^[39]^

RDW is a reliable anisocytosis index used in the differential diagnosis of micro- and normocytic anemias. In addition, studies are showing a statistically significant correlation with HbA1c.^[40,41]^ In the present study, the relationship between HbA1c and RDW-CV was found to be statistically significant, and significant differences were observed between RDW-CV values according to controlled diabetes and uncontrolled diabetes groups. Uncontrolled diabetes can cause functional and structural changes in the red blood cell (RBC)’s hemoglobin molecule and the cytoplasmic environment. The chronic inflammatory process associated with diabetes can affect erythropoiesis and increase RDW by reducing the half-life of erythrocytes.^[33]^

## 5. Limitations

The study has a retrospective design, meaning that the data were reviewed retrospectively. Such a design may have less evidence than a prospective study and may impose limitations on determining causal relationships. The study includes only patients who applied to the outpatient clinic of a single tertiary research hospital. This situation may limit the generalizability of the results and may not reflect the characteristics of patients from different centers.

## 6. Conclusion

The results showed that there were statistically significant differences between NLR, MPV, WBC, MCV, age, and HbA1c levels. Increasing HbA1c levels are generally associated with an increase in these parameters. Also, high HbA1c levels are often associated with increased CRP levels. This is important for determining the risk of complications in diabetic patients and protecting target organs. Lastly, a significant decrease in MCV levels with increasing HbA1c levels was found in our study. This suggests that the evaluation of RBCs in routine checks of diabetic patients may indicate glycemic control. These findings could be valuable in the early detection of complications. However, further prospective studies are needed.

## Author contributions

**Conceptualization:** Kemal Aygün, Olgu Aygün, Özden Gökdemir.

**Data curation:** Kemal Aygün, Özden Gökdemir.

**Formal analysis:** Olgu Aygün, Özden Gökdemir.

**Investigation:** Ayça Asma Sakalli, Özden Gökdemir.

**Methodology:** Kemal Aygün, Halime Seda Küçükerdem, Olgu Aygün, Özden Gökdemir.

**Software:** Olgu Aygün.

**Supervision:** Kemal Aygün, Halime Seda Küçükerdem, Özden Gökdemir.

**Writing – original draft:** Ayça Asma Sakalli, Olgu Aygün.

**Writing – review & editing:** Kemal Aygün, Halime Seda Küçükerdem, Olgu Aygün.
